# Genetic Analysis of cagA and vacA Genes in Helicobacter Pylori Isolates and Their Relationship with Gastroduodenal Diseases in the West of Iran

**DOI:** 10.5812/ircmj.3732

**Published:** 2013-05-05

**Authors:** Negar Souod, Mohammad Kargar, Abbas Doosti, Reza Ranjbar, Meysam Sarshar

**Affiliations:** 1 Young Researchers and Elite Club, Jahrom Branch, Islamic Azad University, Jahrom, IR Iran; 2 Department of Microbiology, Jahrom Branch, Islamic Azad University, Jahrom, IR Iran; 3 Department of Biotechnology, Shahrekord Branch, Islamic Azad University, Shahrekord, IR Iran; 4 Molecular Biology Research Center, Baqiyatallah University of Medical Sciences, Tehran, IR Iran

**Keywords:** Helicobacter Pylori, VacA Protein, Helicobacter Pylori, CagA protein, Helicobacter Pylori

## Abstract

**Background:**

Helicobacter pylori have different virulence factors which are associated with several gastroduodenal diseases; however, this association is variable in different geographical regions. Data of genotypes of Iranian H. pylori isolates are few.

**Objectives:**

The aim of the current study was to investigate the cagA/vacA genotypes of Helicobacter pylori isolates and determine the relationship between these genotypes with respect to different gastric disorders in patients of Chaharmahalo Bakhtiarian.

**Materials and Methods:**

In this cross-sectional study, gastric biopsies were taken from 200 patients with gastrodoudenal diseases. Histopathological features were recognized by specialist. The samples were subjected to PCR for detection and identification of ureC, cagA and vacA genes.

**Results:**

The frequency of the vacA genotypes, sa1/m1, s1a/m1b, s1a/m2, s1b/m1a, s1b/m1b, s1b/m2, s1c/m1a, s1c/m1b, s1c/m2, s2/m1a, s2/m1b and s2/m2 were 27(6.6%), 8(4.3%), 45(28.04%), 7(3.7%), 5(2.5%), 10 (6.1%), 12 (7.4%), 4 (2.5%), 18(11%), 6(3.7%), 0 and 22(13.5%) respectively. The cagA gene was detected in 92% of strains. Based on our findings, it seemed that cagPAI and vacA s1 genotypes were associated with some gastric disorders in patients with H. pylori. In this region, the isolates carrying s1a/m2 were the most prevalent.

**Conclusions:**

We found considerable relationship between s1a/m1a, s1a/m2, s2/m2 and s1c/m1a and some gastric disorders. Further studies about the role of H. pylori virulence factors and gastric disorders were recommended.

## 1. Background

Helicobacter pylori is major causes of chronic gastritis. They are also involved in the pathogenesis of several diseases including gastric and duodenal ulcers, gastric adenocarcinoma and mucosa-associated lymphoid tissue lymphoma (1). Several H. pylori virulent genes have been identified to contribute to the severity of these diseases. The pathogenicity island (PAI) is the most important virulent factor of the bacterium that encodes a type IV secretion apparatus. The cagA gene is located at the end of the cag PAI (2-5). Another important virulence factor of H. pylori is a vacuolating cytotoxin (VacA), which is associated with injury of epithelial cells. The vacA gene is present in nearly all strains of H. pylori but it is polymorphic, comprising variable signal regions (type s1 or s2) and mid-regions (type m1 or m 2). The type s1/m1 vacA strains causes more epithelial cell damage than type s1/m2, whereas type s2/m2 and the rare s2/m1 are non-toxic due to the presence of a short 12-residue hydrophilic extension on the s2 form (3, 6). The s-region is classified into s1 and s2 types and the m-region into m1 and m2 types. The s1 type is further classified into s1a, s1b and s1c subtypes, and the m1 into m1a and m1b subtypes. The mosaic combination of s and m-region allelic types determines the particular cytotoxic and, consequently, the pathogenicity of the bacterium (7, 8). There is a geographical variation in the vacA genotypes (9, 10). For example, studies have consistently shown that vacA s1a strains predominate in northern Europe, s1b in Central and South America, Spain and Portugal, s1a and s1b in the USA and s1c in East Asia (10). These differentiations may cause variations in the prevalence of gastric diseases in these areas. Thus, existing data are contradictory and cannot explain the pathogenic role of H.pylori in the development of different gastric diseases. Furthermore, it might be useful to know the genetic diversity of H.pylori strains in Chaharmahalo Bakhtiari, one of the risky provinces of Iran.

## 2. Objectives

The aim of the current study was to investigate the cagA/vacA genotypes of Helicobacter pylori isolates and determine the association between these genotypes with different gastric disorders in Chaharmahalo Bakhtiarian patients.

## 3. Materials and Methods

### 3.1. Collection of Patient Samples 

From June to November 2009, 200 consecutive patients with dyspeptic symptoms attending the endoscopy suite of gastroenterology section of Hospital of Shahrekord University of Medical Sciences (SUMS) enrolled in the study. The questionnaires, including medical history and demographic data, were recorded for each patient. All studied patients signed an informed consent form before endoscopy and declared their willingness to allow the application of their anonymous data for research purposes. For each patient, two biopsy specimens were taken from the antrum using a disinfected endoscope. One piece of each specimen was examined by Rapid Urease Test (RUT) for detection of H. pylori. RUT was performed with a Gastro urease kit (Baharafshan, Iran). The second piece from positive samples in RUT was placed in 0.1 ml of sterile saline solution and was sent to Biotechnology Research Center of Islamic Azad University, Shahrekord Branch for further studies.

### 3.2. Genomic DNA Extraction and Polymerase Chain Reaction

DNA was isolated from biopsy specimens using Genomic DNA purification kit (DNPTM, CinnaGen, Iran) according to the recommendations of manufacture Primers sequences used for the PCR include as follows: ET-2U (5'-CCCTCACGCCATCAGTCCCAAAAA-3') and ET-2L (5'-AAGAAGTCAAAAACGCCCCAAAAC-3') ( 4 ). Primers used for PCR assays of vacA and cagA genes are listed in (Table 1) ( 11 , 12 ).

**Table 1.** Primers Used for PCR Analysis of vacA and cagA Genes (, )

Region	Primer	Sequence (5'-3')	Size and location of PCR Product
**ureC (glmM)**	GlmM1-R GlmM1-F	GCTTACTTTCTAACACTAACGCGC GGATAAGCTTTTAGGGGTGTTAGGGG	296bp
**s1a**	vacA s1a-F VA1-R	CTC TCG CTT TAG TAG GAG C CTG CTT GAA TGC GCC AAA C	213 bp (843-1055)
**s1b**	SS3-F VA1-R	AGC GCC ATA CCG CAA GAG CTG CTT GAA TGC GCC AAA C	187 bp (869-1055)
**s1c**	vacA s1c-F VA1-R	CTC TCG CTT TAG TGG GGY T CTG CTT GAA TGC GCC AAA C	213 bp (843-1055)
**s2**	SS2-F VA1-R	GCT AAC ACG CCA AAT GAT CC CTG CTT GAA TGC GCC AAA C	199 bp (433-631)
**m1a**	VA3-F VA3-R	GGT CAA AAT GCG GTC ATG G CCA TTG GTA CCT GTA GAA AC	290 bp (2741-3030)
**m1b**	VAm-F3 VAm-R3	GGC CCC AAT GCA GTC ATG GA GCT GTT AGT GCC TAA AGA AGC AT	291 bp (2741-3031)
**m2**	VA4-F VA4-R	GGA GCC CCA GGA AAC ATT G CAT AAC TAG CGC CTT GCA	352 bp (976-1327)
**cagA**	cagA-U cagA-L	GGA ATA CCA AAA ACG CAA AAA CCA CCC CAC AAT ACA CCA GCA AAA CT	300bp

DNA samples from H. pylori (D0008, Genekam, Germany) were used as a positive control of cagA and vacA genes, and sterile distilled water was used as a negative control. PCR was done in 20 µL (for H. pylori) or 25 µL (for vacA and cagA) of total reaction volume containing 1.5 mM MgCl2 (2.0 mM for cagA), 50 mM KCl, 10 mM Tris-HCl (pH 9.0), 0.1% Triton X-100, 200 µM dNTPs each (Fermentas), 0.4 µM primers, 0.3 U of Taq DNA polymerase (Fermentas), and 2 µL (40-260 ng/µL) of DNA. PCR was performed in a DNA Thermal Cycler (Eppendrof Mastercycler 5330, Eppendorf-Nethel-Hinz GmbH, Hamburg, Germany), with 40 cycles for ET2 primer and 35 cycles for vacA and cagA primers. Each cycle consisted of denaturation at 95°C/45 seconds; annealing at 59°C/30 seconds for ET2, 52°C/45 seconds for vacA, and 58°C/45 seconds for cagA; and extension at 72°C/45 seconds (11). There was another time extension (6 minute) at 72°C. PCR products were visualized by electrophoresis in 1% agarose gel, stained with ethidium bromide, and examined under ultraviolet illumination.

### 3.3. Statistical Analysis

The data were analyzed by SPSS software (Version 17.spss Inc, USA) and P value was calculated using Chi-square and Fisher's exact tests to find the significant relationship. P value less than 0.05 was statistically significant.

## 4. Results

Out of 200 gastric biopsy specimens, 164 (82%) were confirmed to be H. pylori infection positive by RUT. Of all patients studied, 79 (48.1%) were male and 85 (51.8%) were female, with a mean age of 47 ± 17 years (Range 15 to 88 years old). Sixteen patients (11.8%) had Gastric ulcers, 22 (16.2%) had Duodenal ulcers, 160 (97.5%) had Gastritis, 3 (2.2%) had Gastric cancer and 3 (2.2%) had Duodenit. Possible combinations of vacA s and m regions were determined in Iranian population. Overall 63 samples were classified as vacA s1/m1, 73 samples as s1/m2, 22 as s2/m2 and 6 as s2/m1 genotypes. Out of 135 s1 strains, all the samples were successfully sub-typed using s1a, s1b and s1c specific primers. Among them, 79 (48.1%) were s1a positive, 21 (12.8%) were s1b positive and 35 (21.3%) were s1c positive. In the case of m1 sub-typing, the distribution of m1a and m1b was 31.7% and 9.14% respectively. M2 was found in 58.5% of the cases (Table 2).

**Table 2.** Correlation BetweenvacA Subtypes and Clinical Outcomes

vacA genotypes	G.U^a^ (n = 16), No. (%)	D.U^a^ (n = 22), No. (%)	G.C^a^ (n = 3), No. (%)	C.G^a^ (n = 160), No. (%)	DUO^a^ (n = 3), No. (%)	Total (n = 164), No. (%)
**S1a**	9 (56.2)	11 (50)	1 (33.3)	76 (47.5)	1 (33.3)	79 (48.1)
**S1b**	3 (18.7	1 (4.5)	1 (33.3)	20 (12.5)	1 (33.3)	21 (12.8)
**S1c**	3 (18.7)	3 (13.6)	0	34 (21.2)	0	35 (21.3)
**S2**	1 (6.2)	7 (31.8)	1 (33.3)	28 (17.5)	1 (33.3)	29 (17.6)
**M1a**	4 (25)	9 (40.9)	2 (66.6)	52 (32.5)	0	52 (31.7)
**M1b**	3 (18.7)	3 (13.6)	0	15 (9.3)	0	15 (9.1)
**M2**	9 (56.2)	10 (45.4)	1 (33.3)	92 (57.5)	3 (100)	97 (59.1)
**S1a/m1a**	1 (6.2)	4 (18.1)	1 (33.3)	27 (16.8)	0	27 (16.4)
**S1a/m1b**	2 (12.5)	2 (9.09)	0	7 (4.3)	0	8 (4.8)
**S1a/m2**	6 (37.5)	2 (9.09)	0	41 (25.6)	1 (33.3)	45 (27.4)
**S1b/m1a**	2 (12.5)	0	0	6 (3.7)	0	7 (4.2)
**S1b/m1b**	0	0	0	4 (2.5)	0	5 (3.04)
**S1b/m2**	1 (6.2)	1 (4.5)	1 (33.3)	10 (6.2)	1 (33.3)	10 (6.09)
**S1c/m1a**	1 (6.2)	1 (4.5)	0	12 (7.5)	0	12 (7.3)
**S1c/m1b**	1 (6.2)	1 (4.5)	0	4 (2.5)	0	4 (2.4)
**S1c/m2**	1 (6.2)	1 (4.5)	0	18 (11.2)	0	18 (10.9)
**S2/m1a**	0	1 (4.5)	1 (33.3)	6 (3.7)	0	6 (3.6)
**S2/m1b**	0	0	0	0	0	0
**S2/m2**	1 (6.2)	6 (27.2)	0	22 (13.7)	1 (33.3)	22 (13.4)

^a^Abbreviation: C.G, Chronic gastritis; D.U, Duodenal ulcer; DUO, Duodenit; G.C, Gastric cancer; G.U, Gastric ulcer

One hundred fifty one (92%) out of 164 ureC- positive samples carried cagA gene. One hundred twenty eight (78.04%) strains with vacA s1 genotype were cagA positive while 23 (14.02%) strains with vacA s2 genotype were cagA positive, indicating that the presence of cagA gene was significantly associated with the vacA s1 genotype (P = 0.004). In particular, most samples (95.5%) with the vacA s1/m2 genotypes were cagA positive. Also, the prevalence of cagA gene was not related to the clinical outcomes (Table 3).

**Table 3.** Correlations BetweencagA Status and Clinical Outcomes

Patients groups	H. pylori-positive (n = 164), No. (%)	cagA-negative (n = 13), No. (%)	cagA-positive (n = 151), No. (%)
**G.U ** ^a^	16 (9.7)	2 (15.3)	14 (9.2)
**D.U ** **^a^**	22 (13.4)	2 (15.3)	20 (13.2)
**G.C ** **^a^**	3 (1.8)	1 (7.6)	2 (1.3)
**C.G ** **^a^**	160 (97.5)	147 (94.2)	12 (7.5)
**DUO ** **^a^**	3 (1.8)	0	3 (1.9)

^a^Abbreviation: C.G, Chronic gastritis; D.U, Duodenal ulcer; DUO, Duodenit; G.C, Gastric cancer; G.U, Gastric ulcer

## 5. Discussion

H. pylori are one of the most genetically diverse bacterial species which may be involved in the complex variety of gastro duodenal diseases in infected patients all over the world (13-16). The geographic prevalence of distinct H. pylori genotypes remains largely unknown (15). For instance, in Japan, South America, Turkey and Pakistan, the prevalence is more than 80%, while in Scandinavia and England, the prevalence is between 20% and 40% (3). The prevalence of this bacterium in Iran is 60-90%, indicating that Iran is a highly risky region for H. pylori infection. The prevalence of this bacterium was 82% in our study indicating that our findings are consistent with previous reports in Iran (16-18). The vacA genotypes show considerable variability in different geographic regions (3). According to our results, 80% of samples had vacA s1a, b, c, also m2 genotypes and s1a/m2 was predominant in H. pylori isolates. This finding is somewhat similar to Europe and North America, where vacA s1a, s1b and m2 are predominating too. Our isolates were similar to those isolated from East Asian isolates where s1c is predominant (15). This study showed that Iranian H. pylori isolates are very diverse in genotype and contain the East and the West elements. H. pylori strains concluding cagA gene are more virulent than cagA-negative strains (3). The prevalence of cagA-positive H. pylori varied from one geographic region to another, e.g., 97% in Korea, 94% in Malaysia, 90% in China, 78% in Turkey and 53% in Kuwait (19). In this study, we found cagA gene in 92% of the H. pylori-positive population. This finding did not demonstrate the role of cagA as predictive marker for increased virulence feature of H. pylori, because of the high positivity of this gene in all H. pylori isolates. A strong association between the cagA and vacA status and peptic ulcer disease has been reported (20-22). Beil et al. suggested that the increased inhibitory effect of cagA-positive, cytotoxin-producing strains on mucin synthesis could be considered as a possible mechanism which is responsible for the increased risk of developing peptic ulceration with these H. pylori strains (9). Gzyl et al. found that cagA gene correlated with active gastritis in infected children and adults. They also found that the majority of H. pylori strains carrying s1/m2 vacA alleles were responsible for the higher levels of cytotoxin production (10). Our data were in agreement with those of Gzyl et al. and Beil et al., which suggest that H. pylori strains with cagA and vacA s1 genotypes are associated with more severe gastritis (9, 10). Investigator's opinions about the association between vacA genotypes and gastric disorders were different. For example, in Iran, Jafari et al. found no correlation between them, (3) whereas Mohammadi et al. and Molaei et al. found that s1a allele were associated with more severe inflammation (15, 23). As results showed, we found an association between some diseases and some vacA genotypes but we couldn’t introduce any allele as a marker of a disease. For instance, s2m2 strains that are non-toxigenic in most regions of the world and are associated with NUD diseases were surprisingly more prevalent in PUD than in NUD patients and had direct association with duodenal ulcer in our evaluation. In Thailand, Japan, Korea, Colombia and America, no association had been found (21) whereas in Cuba, Lebanon, Hung Kung, China and most of the European countries, a significant association between s1 allele and PUD diseases had been reported (20, 21, 24, 25). Data analysis revealed a significant association between Duodenal ulcer and s1a/m1a (P = 0.04), s1a/m2 (P = 0.02) and surprisingly s2m2 (P = 0.05) genotypes. We observed an association between Gastritis diseases and s1c/m2 (P = 0.01). We didn't find any relationship between other diseases with regard to vacA genotypes. In conclusion, we found that the cagA-positive s1/m2 H. pylori were dominant genotypes in the patients under study. The cagA gene positivity rate was probably not closely associated with severity of the disease. H. pylori strains including vacA s1 genotype were associated with more severe gastritis.
